# Both Adaptability and Endophytic Bacteria Are Linked to the Functional Traits in the Invasive Clonal Plant *Wedelia trilobata*

**DOI:** 10.3390/plants11233369

**Published:** 2022-12-04

**Authors:** Ying-Hao Mei, Xu Li, Jian-Yu Zhou, Fang-Li Kong, Shan-Shan Qi, Bin Zhu, Misbah Naz, Zhi-Cong Dai, Dao-Lin Du

**Affiliations:** 1 School of Emergency Management, Jiangsu University, Zhenjiang 212013, China; 2 School of the Environmental and Safety Engineering, Jiangsu University, Zhenjiang 212013, China; 3 School of the Agricultural Engineering, Jiangsu University, Zhenjiang 212013, China; 4 Department of Biology, University of Hartford, West Hartford, CT 06117, USA; 5 Jiangsu Collaborative Innovation Center of Technology and Material of Water Treatment, Suzhou University of Science and Technology, Suzhou 215009, China

**Keywords:** environmental adaptation, endophytic diversity, functional traits, invasive plant, *Wedelia trilobata*

## Abstract

The role of the interactions between endophytes and host plants is unclear in invasive plants from different geographical latitudes. In this study, we aimed to explore the relationship between endophytic microbes and the functional traits of the invasive plant *Wedelia trilobata*. We explored the relationship between endophytes and the clonal growth traits of the invasive clonal plant *Wedelia trilobata* from different geographical latitudes using high-throughput sequencing technology and a common garden-planting experiment. We found that: (1) Different *W. trilobata* populations had similar endophytic fungi but different endophytic bacteria. However, no latitudinal variation pattern of the overall microbial community was found; (2) plant clonal growth performance (i.e., spacer length) was significantly correlated with endophytic bacterial diversity but not fungal diversity; and (3) the latitudinal variation pattern of the plant clonal growth performance of *W. trilobata* populations was found in pre-cultivated (i.e., wild) individuals but disappeared in post-cultivated *W. trilobata*. Our results suggest both environmental adaptability and the endophytic bacterial community are linked to the functional traits of the invasive clonal plant *W. trilobata*, and these functional traits tend to increase its invasiveness, which may enhance its invasion success.

## 1. Introduction

Microorganisms and plants in the natural environment are connected in various ways, resulting in a variety of plant–microbe symbiotic interactions. Plant endophytes are generally a group of microorganisms that exist as symbionts in plants, mainly including endophytic bacteria and endophytic fungi, and these microorganisms provide profound benefits for plants through their interactions with the plants [1,2,3]. For example, endophytic bacteria promote plant growth through conducting nitrogen fixation [4], regulating the synthesis of phytohormones [5], and improving the host plant’s resistance to abiotic (e.g., cold, drought, saline-alkali) [6,7] and biotic (e.g., plant diseases) [8] environmental stresses. Invasion of alien plant species, as a consequence of global changes, usually causes significant losses of biodiversity and economy in the invaded regions [9,10,11,12]. Several studies in recent years have connected endophyte traits to invasive plant behavior. Studies [13,14] have proved that endophytes can indeed enhance the ability of invasive plants directly or indirectly. For example, *Spartina alterniflora* not only directly competes with native plants, it also indirectly causes pathogen infection on native plants via endophytic fungi [15]. In addition, endophytic bacteria can promote the production of phytohormones to help plant growth and indirectly influence the functional features of the plant host [16]. The functional traits of invasive clonal plants play an important role in their invasion process, which often makes them more competitive than native plants [17]. Thus, interactions between invasive plants and endophytes have an impact on the ecology, distribution, and variety of flora and wildlife. Over the past ten years, interest has grown in how important these interactions are to ecosystem function by encouraging nutrient intake and changing the defense mechanisms of plants [18].

Despite the fact that diverse geographical regions are connected with endophytes of host plants [18,19,20], the role of endophytes on plant invasion is still unclear, as cultivable endophytes or endophytic communities of invasive plants were explored separately in previous studies. For instance, Fang et al. [21] showed that the community of cultivable endophytic fungi based on tissues within the invasive plant *Ageratina adenophora* changed across geographic areas. Cheng et al. [22] discovered numerous invasive plant populations. A basic group of endophytic bacteria was present in *Senecio vulgaris*. These studies concentrated on endophyte variations without demonstrating the impact of functional variations on plant invasion success. Therefore, it is crucial to understand the link between invasive plants and endophytes because doing so could lead to the development of novel biological methods for controlling invasive plants.

The invasive clonal plant *Wedelia trilobata* (L.) Hitchc., one of the 100 worst invasive alien species in the world [23], is a creeping herb native to tropical Central America and has invaded many areas of the tropics and subtropics. Clonal propagation, with extremely strong phenotypic plasticity and adaptability, is the main mode of reproduction of *W. trilobata* [24]. At present, *W. trilobata* has been further expanding from South China to Central China. We also found that *W. trilobata* successfully expanded northward to the central and northern parts of East China, such as Wenzhou and Taizhou Cities, Zhejiang Province. Current research on *W. trilobata* focuses on resource utilization [25,26,27], allelopathy [28,29,30], clonal propagation strategy analysis [24,31], and environmental factor stress response [32,33,34]. Considerable beneficial effects of tissue-cultivable endophytic bacteria on the clonal growth of *W. trilobata* have been confirmed [2], yet few studies investigate the community composition of the endophytic microorganism community of *W. trilobata* from different populations and its role on the growth performance of this invasive plant. Therefore, exploring the endophytic microbial composition and its association with the growth phenotypes of different populations of *W. trilobata* in the invasion areas is of great ecological significance. The results will provide better understanding in prediction of further invasion and expansion of alien plants.

We investigated the functional characteristics of the corresponding population of *W. trilobata* under a homogeneous growth environment, as well as the endophytic microbial community of four distinct populations of *W. trilobata* from four different provinces in China. We tested the following hypotheses: (1) a latitudinal gradient of the four populations of *W. trilobata* is associated with the pattern of or the difference in endophytic microbial diversity and communities, and (2) the functional traits of *W. trilobata* are related to the geographic pattern of endophytic microbial diversity and communities.

## 2. Results

### 2.1. Endophytic Microbial Diversity and Composition

The rarefaction curve (Figure S1) indicated sufficient sequencing depth. Sequencing of microbial communities generated a total of 0.93 million bacterial sequences (average of 77,590 sequences per sample) and 1.38 million fungal sequences (average of 114,902 sequences per sample). More detailed results are shown in the Supplementary Materials Table S1. These sequences were clustered into 436 and 1987 distinct operational taxonomic units (OTUs) for bacteria and fungi, respectively. In addition, the number of core flora of endophytic bacteria and fungi differed significantly (Figure S3). 

**Figure 1:**
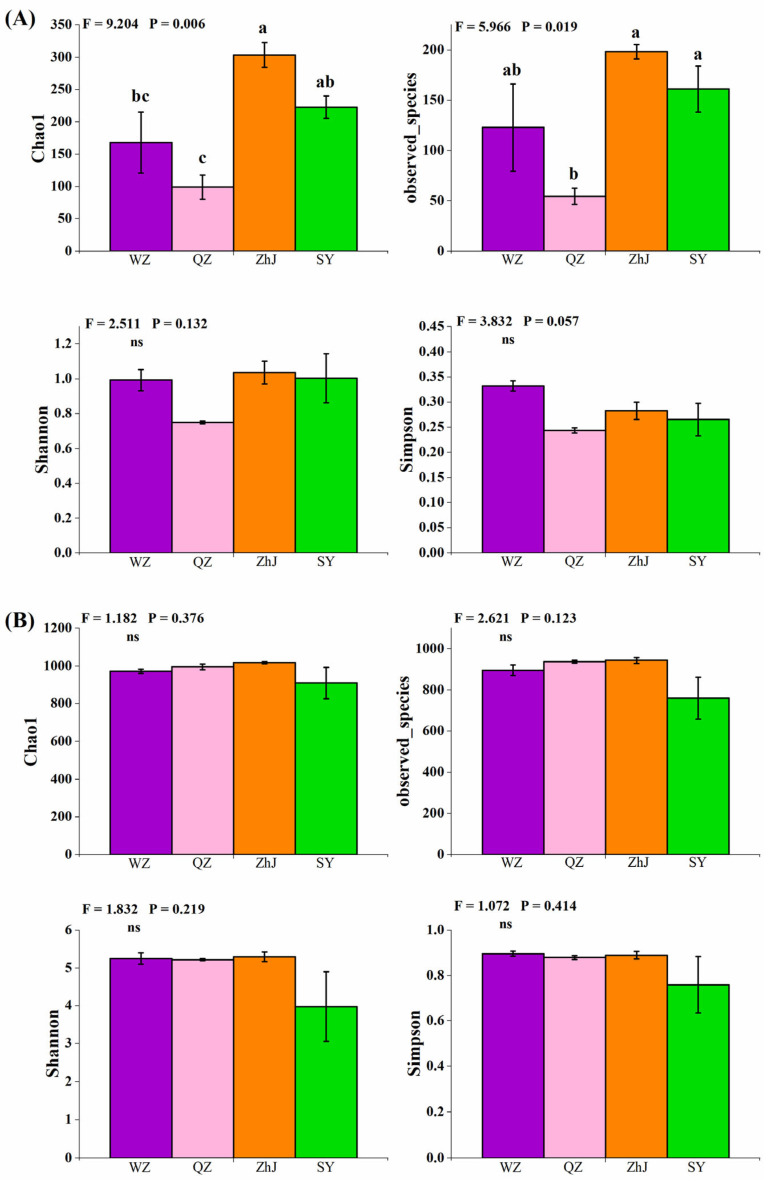
Differences in the α diversities of endophytic bacterial (**A**) and fungal (**B**) communities of *W. trilobata* from different populations (WZ, Wenzhou City; QZ, Quanzhou City; ZhJ, Zhanjiang City; SY, Sanya City). Bars (mean with standard error, *n* = 7) with different lowercase letters represent statistically significant differences (*p* < 0.05). “ns” means no statistically significant difference (*p* > 0.05).

We found that the alpha diversities of endophytic bacterial communities of *W. trilobata* from different populations were significantly different (Figure 1A). The Chao1 index and the observed species of *W. trilobata* from the QZ population were significantly lower than those of *W. trilobata* from other places, while the alpha diversities of *W. trilobata* from ZhJ and SY were similar (Figure 1A). However, the alpha diversities of soil fungal communities did not change significantly among *W. trilobata* from different geographic regions (Figure 1B).

**Figure 2:**
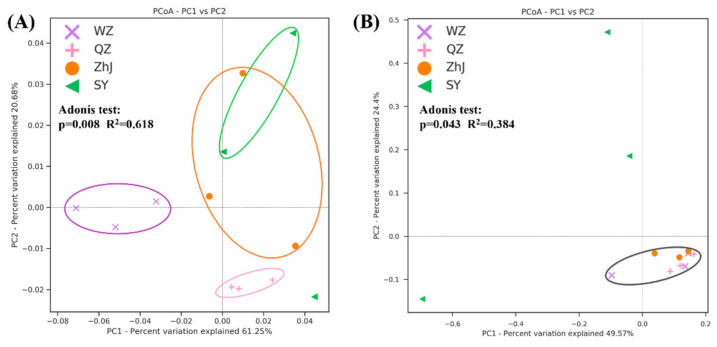
PCoA plots of endophytic bacterial (**A**) and fungal (**B**) communities of *W. trilobata* from different places based on the Bray–Curtis distance (WZ, Wenzhou City; QZ, Quanzhou City; ZhJ, Zhanjiang City; SY, Sanya City).

Beta-diversity analysis showed that the bacterial community compositions of *W. trilobata* from different populations were significantly different (Figure 2A). However, there was little difference in the composition of the fungal community except *W. trilobata* from the SY population (Figure 2B). The community compositions of *W. trilobata* in endophytic bacterial communities from QZ and WZ differed distinctly from the *W. trilobata* from ZhJ and SY, according to the Bray–Curtis distances (Figure 2A). *W. trilobata* from the ZhJ and SY populations had similar community structures for bacteria and fungi. In addition, the β diversity of *W. trilobata* in endophytic bacterial communities from QZ differed significantly from that of *W. trilobata* in endophytic bacterial communities from the most northern population of WZ (Figure 2A).

**Figure 3:**
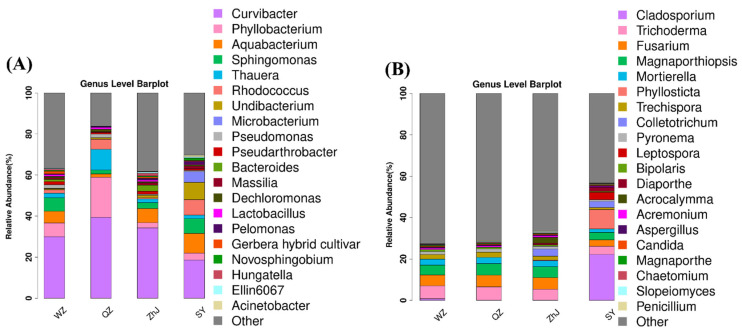
The relative abundances of endophytic bacterial (**A**) and fungal (**B**) communities at the genus level (WZ, Wenzhou City; QZ, Quanzhou City; ZhJ, Zhanjiang City; SY, Sanya City); ‘Other’ indicates OTUs that have not been annotated.

Although the compositions of core endophytic bacteria of *W. trilobata* were similar at the phylum level (Figure S4), the relative abundances of endophytic bacteria in the stems of *W. trilobata* varied greatly among different populations at the genus level. Plant samples from different populations were found to be dominated by bacteria from the genera *Curvibacter*, *Phyllobacterium*, *Aquabacterium,* and *Sphingomonas* (Figure 3A). Compared with *W. trilobata* from other populations, the relative abundances of *Curvibacter*, *Phyllobacterium,* and *Thauera* in the QZ population were significantly greater than that of other populations, while the relative abundances of *Sphingomonas* and *Aquabacterium* were significantly lower than that of other populations (Figure 3A). Similarly, Circos analysis at the phylum level showed differences in the endophytic core flora of *W. trilobata* (Figure S5).

The relative abundance of endophytic fungi in the stems of *W. trilobata* from the southernmost population SY was significantly different from that of other populations. However, there were no significant differences in the relative abundances of fungi in other populations of *W. trilobata* (Figure 3B). Different from the observed bacterial community, there were no significant differences in the fungal community structure. The most abundant fungi found in *W. trilobata* were *Trichoderma, Fusarium, Magnaporthiopsis,* and *Mortierella* (Figure 3B).

**Figure 4:**
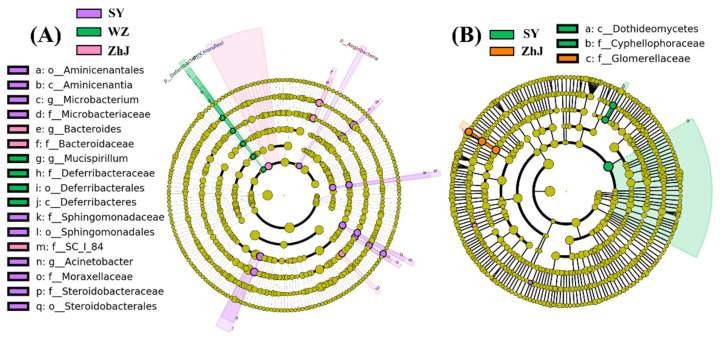
The LEfSe analysis of endophytic bacteria (**A**) and fungi (**B**) in *W. trilobata.* (No biomarkers were found in the bacterial community of QZ.)

LEfSe analyses were implemented to assess whether any statistically significant differences occurred in the taxon abundance of endophytic microorganisms and to check the biological relevance of the species in different populations of *W. trilobata* (Figure 4). Specifically, *Deferribacterales* and *Mucispirillum* were the main biomarkers in endophytic bacteria of WZ; *Bacteroides*, *Bacteroidaceae*, *Chloroflexi,* and *SC_I_84* were the main biomarkers in endophytic bacteria of ZhJ; and *Microbacterium*, *Sphingomonadales*, *Aegiribacteria*, *Steroidobacteraceae*, *Moraxellaceae*, *Acinetobacterjohnsonii*, *Acinetobacter,* and *Aminicenantales* were the main biomarkers in endophytic bacteria of SY (Figure 4A). In addition, *Microascus* and *Agrocybe* were the only biomarkers in WZ and QZ endophytic fungi, respectively; *Glomerellaceae*, *Colletotrichum,* and *Colletotrichum_brevisporum* were the main biomarkers in endophytic fungi of ZhJ; *Dothideomycetes* and *Cyphellophoraceae* were the main the biomarkers in endophytic fungi of SY (Figure 4B).

### 2.2. Plant Phenotypic Growth

**Figure 5:**
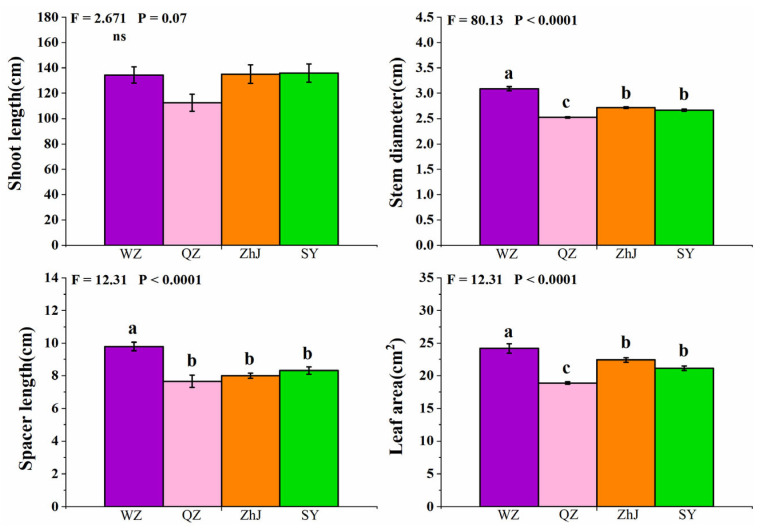
Shoot length, stem diameter, spacer length, and leaf area of different populations of *W. trilobata* (WZ, Wenzhou City; QZ, Quanzhou City; ZhJ, Zhanjiang City; SY, Sanya City). Bars (mean with standard error, *n* = 7) with different lower case letters represent statistically significant differences (*p* < 0.05).

We counted the plant phenotypic growth in the common garden. Stem diameter, spacer length, and leaf area of the northernmost population WZ were significantly higher than those of other populations, and the stem diameter and leaf area index of the QZ population were significantly lower than other populations. However, there were no significant differences between the ZhJ and SY populations in any index (Figure 5).

### 2.3. Correlation Patterns between Microbial Diversity and Growth Indices of W. trilobata

Pearson correlation showed that the stem diameter and spacer length of *W. trilobata* in the pre- and post-cultivated environments were significantly positively correlated with the Simpson diversity index of endophytic bacteria (Table 1; *p* < 0.05). There was a correlation between the growth indicators of pre-cultured *W. trilobata* and its geographical location (Table 1; *p* < 0.05); however, the correlation was not found after cultivating it in the common environment.

## 3. Discussion

The results of this study found that: (1) the endophytic bacterial biodiversity differed significantly among *W. trilobata* populations, but the fungal biodiversity did not. However, no latitudinal variation pattern of the overall microbial community was found. (2) Plant clonal growth performance was significantly correlated with endophytic bacterial diversity but not fungal diversity. (3) The latitudinal variation patterns of plant clonal growth performances of *W. trilobata* populations were found in pre-cultivated (i.e., wild) individuals but disappeared in post-cultivated *W. trilobata*. In summary, our results suggested that both environmental adaptability and the endophytic bacterial, but not fungal, community could affect functional traits in invasive clonal *W. trilobata*.

### 3.1. Geographical Changes of Endophytic Microbe and Clonal Growth Performances in Different Populations of W. trilobata

In general, the sorts of endophytic bacteria that a plant tissue hosts can be determined by the genotype of the host plant, by geographic location, and even by specific plant parts [35]. For example, different tissue parts and geographical locations of the invasive *Ageratina adenophora* affected the composition of the endophytic fungal community [21]. In addition, the endophytic bacteria in *Senecio vulgaris* among different geographical locations were also significantly different [22]. Similarly, we also found that there were significant differences in endophytic bacteria of *W. trilobata* among different geographical locations, although no significant differences were found in the compositions of endophytic fungi (Figure 1A,B). It can be said that the differences in the composition of endophytic bacteria caused by geographical differences among different populations of *W. trilobata* are obvious. 

Geographical location has a far-reaching impact on the growth of plants, not only on the phenotypic growth of plants [36] but also on the composition of plant microbial communities [37]. Numerous studies have demonstrated that the diversity of endogenous microorganisms is influenced by the environmental variations brought on by geographic variances [38,39]. However, the results of this study’s correlation analysis revealed that there were no meaningful associations between variations in *W. trilobata* populations’ microbial diversities and latitudes (Table 1). In a prior investigation, we discovered that *W. trilobata* exhibited a high level of local adaptation to boost the success of its invasion [24]. In this study, the correlation between the growth indicators of pre-cultured, not post-cultured, *W. trilobata* and its geographical location (Table 1) may therefore represent adaptive differences among populations, which could contribute to the invasion success of *W. trilobata*.

### 3.2. The Endophytic Microbial Composition May Contribute to the Clonal Phenotypic Growth of Invasive W. trilobata

Generally, clonal characteristics can be divided into clonal integration and clonal growth. Clonal growth is an asexual reproduction method used by plants that produces genetically identical but possibly independent ramets. This method allows a species to procreate and avoid inbreeding depression, even in the presence of modest initial population levels [40]. It also offers competitive advantages, such as the ability to nurse new ramets, opportunities of scale and division of labor through resource sharing between ramets, and pre-emption of resources through spatial occupation, as well as avoidance of the costs and risks involved in sexual reproduction [41]. For instance, stolons and rhizome internodes of clonal plants can function as places to store resources, such as glucose, which represents a poor method to deal with stressful situations and boosts the survival and re-growth ability of clonal plants, especially following disturbances. Clonal plants are able to withstand harsh conditions and successfully occupy new habitats or endure disruptions thanks to the mobilization of carbohydrates stored in clonal organs such as stolons or rhizomes [42,43,44,45].

The higher performance of growth, competitiveness, and stress resistance is critical for alien plants’ invasion success in the early colonization stage [46]. The clonal growth traits can help plants adapt to new environments more quickly and enable alien clonal plants to colonize and compete successfully in a wide range of habitats [47] by mediating environmental stresses and sharing resources between ramets (i.e., stolon or rhizome structures) [48]. As a successful creeping clonal plant invader [26,34], *W. trilobata* may be subject to environmental stresses in the new areas during the colonization stage of its invasion. In the homogeneous garden environment, stem diameters, spacer lengths, and leaf areas of the WZ population were all at the highest level (Figure 5). Stem diameter can reflect the resource storage capacity of cloned plants [47], so thicker stems mean more resource storage in the plant stems; invasive plants with slender stems may have weak viability in the new environment. Spacer length of cloned plants can often reflect the space occupation ability of invasive plants in a new habitat [45] and predict the spatial structure pattern of their growth [49]. Several studies have shown that some plants have successfully invaded certain environments due to higher leaf area [50,51], which can often enable invasive species to obtain more ground resources and grow rapidly [31]. Considering that the climates of the three other populations outside Wenzhou are warm, and cold weather is the main limiting factor for the expansion of *W. trilobata*, higher levels of stem diameters, spacer lengths, and leaf areas of the WZ population area therefore more conducive to the expansion of invasive clonal *W. trilobata* in a new cold habitat. Our findings suggest that the clonal phenotypic growth difference in *W. trilobata* may be due to different climatic and environmental conditions. Specially, the colder climate of the WZ population may shape the different clonal phenotypic growth for *W. trilobata*.

Endophytic bacteria have been shown to provide several beneficial effects on their plant host directly or indirectly. They can benefit plants directly to assist plants in getting nutrients and to improve plant growth by modulating growth-related hormones, which can help plants grow better under normal and stressed conditions [52]. By deterring phytopathogens through the synthesis of antibiotics and lytic enzymes, making nutrients unavailable to the pathogens and priming plant defense mechanisms, endophytic bacteria indirectly promote plant growth while defending the plants from future pathogen attacks [53]. In this study, the endophytic bacterial diversity of different populations of *W. trilobata* was significantly and positively correlated with the clonal phenotypic growth of *W. trilobata* (Table 1). The lack of endophytic bacterial diversity in plant tissues may lead to the weakening of phenotypic growth [16]. On the contrary, diverse endophytic bacteria enable *W. trilobata* to gain advantages in growth phenotypes, such as spacer length [2], which make its invasive expansion more efficient. Thus, we speculate that the low bacterial diversity in the tissue may lead to poor phenotypic growth of *W. trilobata* from the QZ population (Figure 1). 

Endophytic bacteria can also enhance nutrient accumulation and metabolism of host plants by producing growth-regulating phytohormones [54,55]. In addition to the lack of diversity, the abundances of some important endophytic bacteria of *W. trilobata* from QZ, such as *Sphingomonas* and *Pseudarthrobacter*, was significantly lower than those of the WZ population, which was shown to play an active role in plant growth (Figure 3) [56,57]. In addition, we found that the abundances of most of the endophytic bacteria with a positive role in plant growth in the WZ population were at high levels, such as *Sphingomonas*, *Pseudarthrobacter,* and *Novosphingobium* [58]. Therefore, we believe that the difference in endophytic bacterial community abundance also had a potential impact on the phenotypic growth of *W. trilobata*, although the specific mechanism of beneficial endophytes remains unclear.

In short, in addition to environmental adaptability, it is endophytic bacteria instead of endophytic fungi that mainly also affect the phenotypic growth of *W. trilobata* among different populations. However, future studies need to be further improved as follows: (1) long-term monitoring experiment combining environmental data on the different geographic environments associated with plant growth; (2) pure endophytes should be isolated from different plant tissue parts to explore the bioactivity of endophytes on plant invasion.

## 4. Materials and Methods

### 4.1. Plant Materials and Pretreatment

To assess whether there is a geographic pattern in endophytic microbial diversity and communities and whether these differences are associated with the growths of different populations of *W. trilobata*, stems of *W. trilobata* from public wastelands of four cities from north to south in China were collected: (1) Wenzhou City (WZ: 27.919617N, 120.698494E), Zhejiang Province; (2) Quanzhou City (QZ: 24.901539N, 118.616325E), Fujian Province; (3) Zhanjiang City (ZhJ: 21.270611N, 110.351982E), Guangdong Province; and (4) Sanya City (SY: 18.28169N, 109.508509E), Hainan Province. We collected complete individual plants of *W. trilobata* from the above cities and recorded the initial growth indicators (i.e., shoot length, stem diameter, spacer length, and stem number; more details can be found in the Supplementary Materials Figure S2), and then sent them to the lab for further cultivation. Stems with similar lengths and thicknesses were selected for the following common garden experiment for phenotypic growth analysis [29] and surface disinfection [59] for endophytic microbial community analysis using high-throughput sequencing of microbial amplicons.

### 4.2. Common Garden Experiment

To assess whether the geographic difference in endophytic microbial diversity and communities contribute to the growth of different populations of *W. trilobata*, a common garden planting experiment was conducted in a greenhouse at Jiangsu University. In the experiment, stem segments from these four populations of *W. trilobata* with similar lengths and thicknesses were selected; each stem segment retained two stem nodes. The stem segments were placed in plastic flowerpots of 90 mm × 60 mm × 80 mm, with one stem segment per pot, seven independent pots for each population of *W. trilobata,* resulting in a total of 28 pots. A mixture of commercial nutrient soil (commercial nutrient soil consisted of different organic matter, with a pH of about 6.5) and vermiculite (5:1 in volume ratio) of 400 g was put in each flowerpot. All the flowerpots were placed in the greenhouse (25 °C/20 °C, day/night average) at Jiangsu University and watered with 100 mL of pure water every day at noon. After 12 weeks of cultivation, all the plants were harvested to measure plant growth indicators (i.e., shoot length, stem diameter, spacer length, and leaf area).

### 4.3. Microbial Community Sequencing and Analysis

First, three stem segments with similar lengths and thicknesses from each *W. trilobata* population as plant tissue samples were selected. Next, in order to retain the endophytes in the plant tissue, the stems of *W. trilobata* from the field were surface-disinfected with 5% sodium hypochlorite one time and rinsed with sterile distilled water three times. The plant tissue samples (three replicates per *W. trilobata* population) were stored on dry ice and then sent to Genepioneer Biotechnologies Co., Ltd. (Nanjing, China) for DNA quantification and qualification. Library construction of endophytic bacteria and fungi and amplicon sequencing were used on the Illumina Novaseq 6000 platform to obtain paired-end (PE) reads. Amplification of the V3–V4 region of the bacterial 16S rRNA genes was completed using the universal primers (341F: 5′-CCTACGGGNGGCWGCAG-3′; 806R: 5′-GGACTACHVGGGTWTCTAAT-3′). The ITS2 region of the fungal rRNA genes was amplified with ITS1 and ITS2 primer (ITS1F: 5′-CTTGGTCATTTAGAGGAAGTAA-3′; ITS2R: 5′-TCCTCCGCTTATTGATATGC-3′). After adding adapters, we used the Illumina Novaseq6000 platform and performed sequencing to obtain 2 × 250 bp paired-end data. Through splicing, a longer sequence was obtained for subsequent analysis.

To make the results of information analysis more accurate and reliable, it was necessary to eliminate the interfering data. First, the original data (deposited in CNGBdb) were spliced and filtered to obtain valid data. Then, OTUs (operational taxonomic units) clustering and species classification analysis were performed based on valid data. According to the OTUs clustering results, species annotations were made for each OTU sequence to obtain corresponding species information and species-based abundance distribution. By using OTU-based abundance and annotation information, we counted the proportion of the sequence number of each sample at each classification level (phylum, class, order, family, genus) to the total sequence number, which can effectively evaluate the species annotation of the sample resolution (the higher the proportion of annotated to genus, the better the OTU annotation effect of the sample), and the species complexity of the sample. (The lower the proportion annotated to the genus, the higher the species complexity of the sample.) Clean reads with the same sequence were first grouped into a single tag, and the abundance (i.e., the number of reads) corresponding to each tag was counted; then, the tags were sorted according to abundance, and the sequences of singletons (corresponding to only one read) were filtered out, which were removed because singletons may be caused by sequencing errors. The clustered sequences were filtered out using a search at 0.97 similarity, and the species were classified in the chimeric OTU; then, the singleton sequences were compared with the representative OTU sequences with a similarity of 0.97. The unaligned sequence would not enter the subsequent analysis, and the sequence on the alignment would be used as one of the OTUs read for follow-up analysis. At the same time, the abundance and alpha diversity calculations of OTUs were analyzed to obtain the species richness and uniformity information in the sample and the common and unique OTUs information among different samples or groups. On the other hand, multiple sequence alignments of OTUs were performed, and a phylogenetic tree was constructed. More details can be found in the Supplementary Materials (Table S1). 

### 4.4. Statistical Analyses

The alpha diversity of the endophytic microorganism was weighed using the succeeding indexes, i.e., Chao1 index, observed species, Shannon diversity, and Simpson dominance. Further, the correlations among the beta diversity estimates of the endophytic microorganism were estimated using the Bray–Curtis algorithm via principal coordinates analysis (PCoA). Through PCoA, dimensionality reduction analysis, and display, the differences in the community structure between different samples or groups were explored. The significance of microbial community variation between treatments was assessed using the Adonis test at α level = 0.05.

Endophytic microbial features in four population were categorized using the linear discriminant analysis (LDA) effect size (LEfSe) method for biomarker discovery, which emphasized the statistical significance and biological relevance. With the normalized relative abundance matrix, the LEfSe method used the Kruskal–Wallis rank-sum test to unearth the features with significantly different abundances between the assigned taxa and performed LDA to estimate the effect size of each feature. A significance level of 0.05 and an effect size threshold of 3 were used for all the biomarkers evaluated in this study.

Differences in the phenotypic growth indices, as well as alpha diversity of endophytic microbial communities, were examined by ANOVA, followed by multiple comparisons via Duncan’s test. The correlation analysis (using the Pearson methods) was performed to analyze the relationships between each phenotypic growth index and alpha diversity of endophytic microbial communities. Statistical analysis processing was completed using IBM SPSS Statistics (version 26.0; IBM Corp., Armonk, NY, USA). The significance level was set at *p* < 0.05.

## Figures and Tables

**Table 1:** Relationships among plant growth performances, geographical latitudes, and diversities of endophytic microbial communities. *p*-values equal to or lower than 0.05 are in bold print.

				Plant Growth Performance								Geographical Latitude
			Pre-Cultivate					Post-Cultivate				
			Shoot Length	Stem Diameter	Spacer Length	Stem Number	Shoot Length	Stem Diameter	Spacer Length	Stem Number	Leaf Area	
**Bacterial**	Chao1 index	r	−0.151	0.1418	−0.195	−0.0411	0.3882	0.1187	0.3078	−0.012	0.372	−0.512
		*p*	0.6384	0.6602	0.5432	0.8990	0.2124	0.7133	0.3304	0.97	0.234	0.089
	Observed species	r	−0.048	0.2336	−0.064	−0.0417	0.3847	0.2136	0.4645	−0.202	0.417	−0.478
		*p*	0.8823	0.4649	0.8438	0.8975	0.2169	0.5051	0.1282	0.53	0.178	0.116
	Shannon diversity	r	0.0521	0.4535	0.2475	−0.1538	0.336	0.3786	0.4867	−0.282	0.446	−0.237
		*p*	0.8723	0.1387	0.4381	0.6331	0.2856	0.2249	0.1086	0.374	0.147	0.458
	Simpson diversity	r	−0.185	**0.776**	**0.702**	−0.542	0.3396	**0.713**	**0.596**	−0.407	**0.636**	0.406
		*p*	0.565	**0.003**	**0.011**	0.0687	0.2801	**0.0093**	**0.041**	0.19	**0.026**	0.191
**Fungal**	Chao1 index	r	−0.304	−0.0951	−0.139	−0.0851	−0.053	−0.0198	0.0321	−0.081	0.0689	0.248
		*p*	0.3371	0.7688	0.6664	0.7926	0.8704	0.9512	0.9211	0.803	0.8314	0.436
	Observed species	r	−0.495	−0.0517	−0.101	−0.2352	−0.186	0.0044	0.0069	−0.143	0.0714	0.418
		*p*	0.1018	0.8732	0.7551	0.4618	0.5625	0.9891	0.983	0.657	0.8255	0.176
	Shannon diversity	r	−0.452	0.0807	0.0735	−0.3209	−0.026	0.1604	0.1581	−0.187	0.1718	0.472
		*p*	0.1399	0.8031	0.8204	0.3091	0.9355	0.6186	0.6236	0.561	0.5934	0.121
	Simpson diversity	r	−0.369	0.0875	0.1297	−0.3018	0.0319	0.173	0.2058	−0.21	0.1606	0.420
		*p*	0.2385	0.7868	0.6879	0.3404	0.9215	0.5908	0.5211	0.511	0.6181	0.174
**Geographical Latitude**		r	r	0.536	**0.64**	**−0.737**	−0.203	0.523	0.328	−0.51	0.285	-
		*p*	*p*	0.73	**0.025**	**0.006**	0.528	0.081	0.299	0.091	0.368	-

## Data Availability

The microbial sequencing data that supported the findings of this study have been deposited into the CNGB Sequence Archive (CNSA) of China National GeneBank DataBase (CNGBdb, https://www.cngb.org/, accessed on 5 September 2022) with accession number CNP0003006. The data presented in this study are available upon request from the corresponding author (e-mail: daizhicong@163.com).

## Supplementary Materials

The following supporting information can be downloaded at: https://www.mdpi.com/article/10.3390/plants11233369/s1, Figure S1: Rarefaction curve of endophytic bacteria (A) and fungi (B) in four populations of *W. trilobata*; Figure S2: Phenotypic growth indexes of four populations of *W. trilobata* collected from different regions; Figure S3: Petal pattern analysis of endophytic bacteria (A) and fungi (B) in four populations of *W. trilobata*; Figure S4: The relative abundances of endophytic bacterial (A) and fungal (B) communities of four populations of *W. trilobata* at the phylum level; Figure S5: Circos analysis of endophytic bacteria (A) and fungi (B) in four populations of *W. trilobata* at phylum level; Table S1: Sequencing evaluation of bacterial and fungal communities in four populations of *W. trilobata*.
