# Supplementary material for: Both Adaptability and Endophytic Bacteria Are Linked to the Functional Traits in the Invasive Clonal Plant Wedelia trilobata
Source: Plants (Basel). 2022 Dec 4;11(23):3369. doi: 10.3390/plants11233369 (PMC9738965; doi:10.3390/plants11233369)
Supplement: Supplementary file 1 [file plants-11-03369-s001.zip › plants-2031155-supplementary.pdf]

plants

Supplementary Materials

Both Adaptability and Endophytic Bacteria Are Linked to the Functional Traits in the Invasive Clonal Plant *Wedelia trilobata*

Ying-Hao Mei [1,2], Xu Li [2], Jian-Yu Zhou [2], Fang-Li Kong [2], Shan-Shan Qi [3], Bin Zhu [4], Misbah Naz [2], Zhi-Cong Dai [1,2,5,*] and Dao-Lin Du [1,2]

1 School of Emergency Management, Jiangsu University, Zhenjiang 212013, China
2 School of the Environmental and Safety Engineering, Jiangsu University, Zhenjiang 212013, China
3 School of the Agricultural Engineering, Jiangsu University, Zhenjiang 212013, China
4 Department of Biology, University of Hartford, West Hartford, CT 06117, USA
5 Jiangsu Collaborative Innovation Center of Technology and Material of Water Treatment, Suzhou University of Science and Technology, Suzhou 215009, China
* Correspondence: daizhicong@163.com

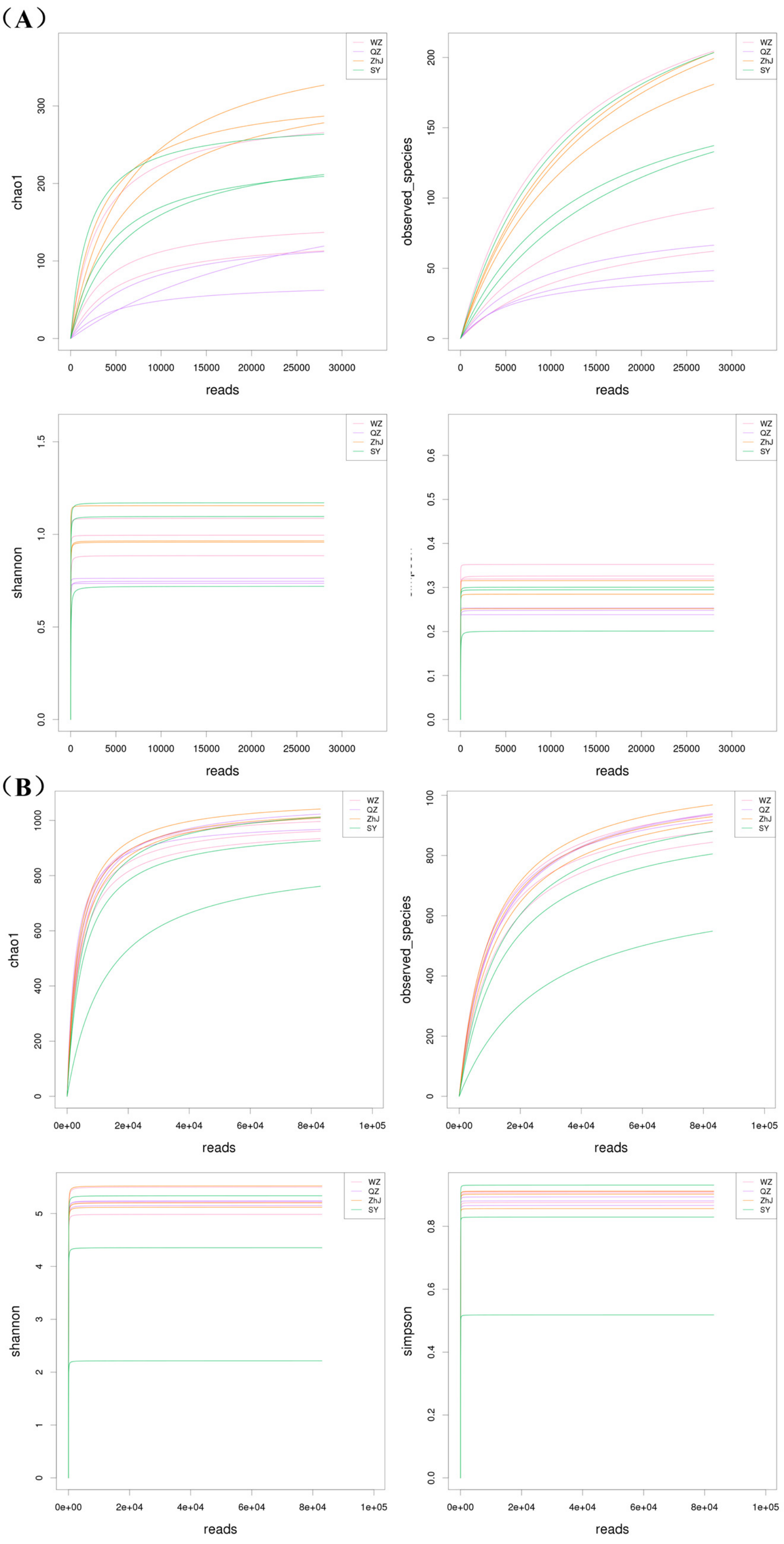

Figure S1: Rarefaction curve of endophytic bacteria (A) and fungi (B) in four populations of *W. trilobata*.

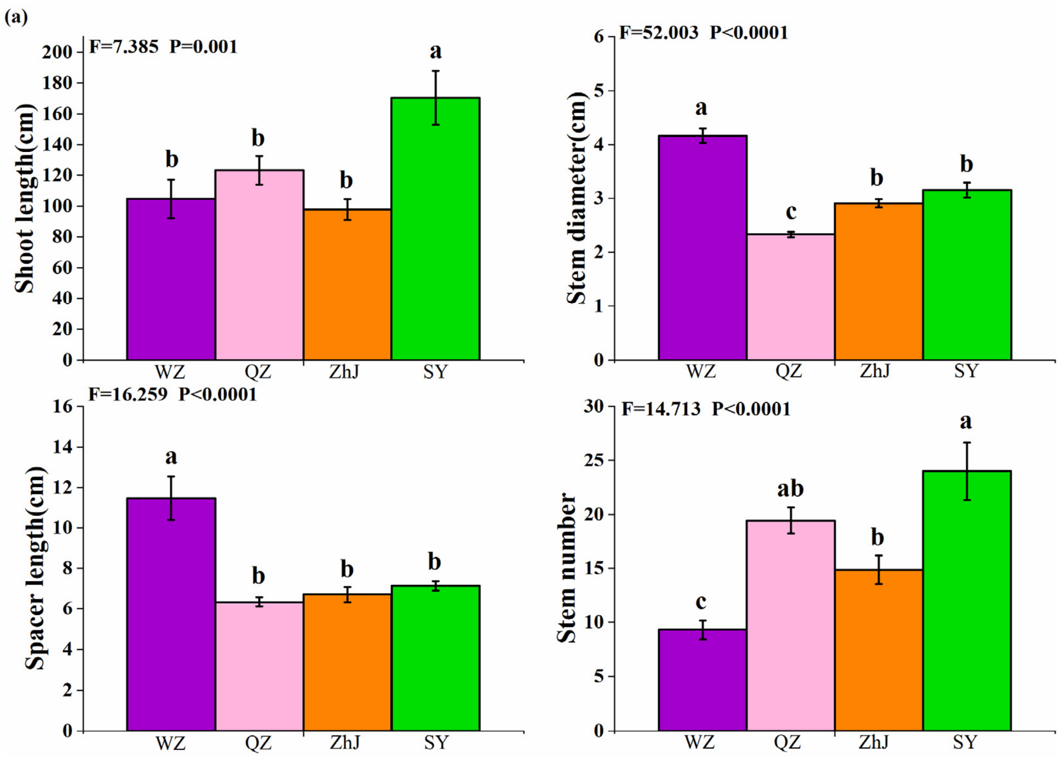

Figure S2: Phenotypic growth index of four populations of *W. trilobata* collecting from different regions (WZ, Wenzhou City; QZ, Quanzhou City; ZhJ, Zhanjiang City; SY, Sanya City). Bars (mean with standard error, n = 7) with different lowercase letters represent statistically significant differences (P ≤ 0.05).

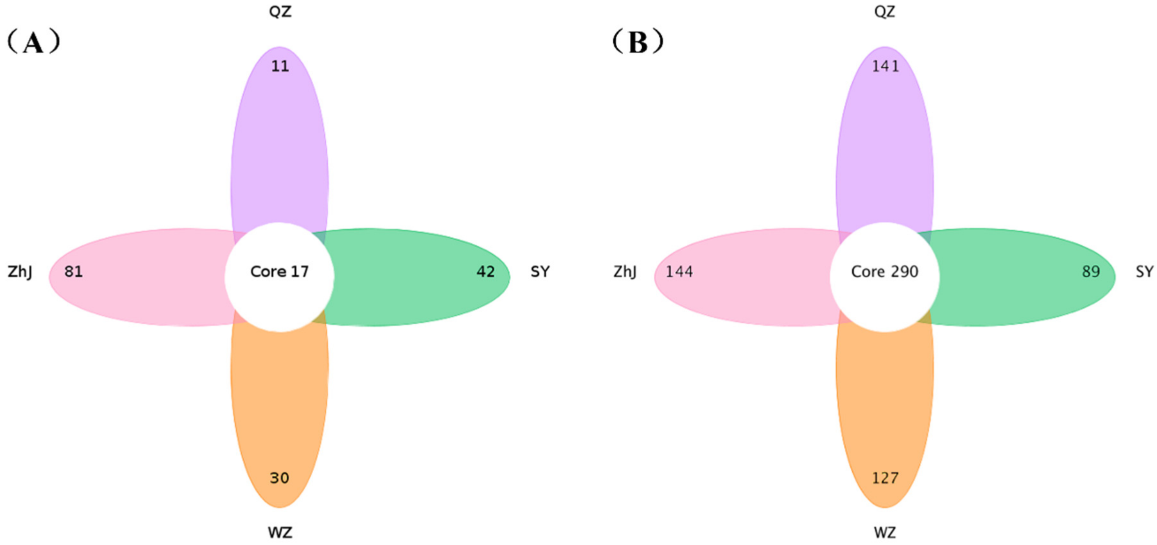

Figure S3. Petal pattern analysis of endophytic bacteria (A) and fungi (B) in four populations of *W. trilobata*.

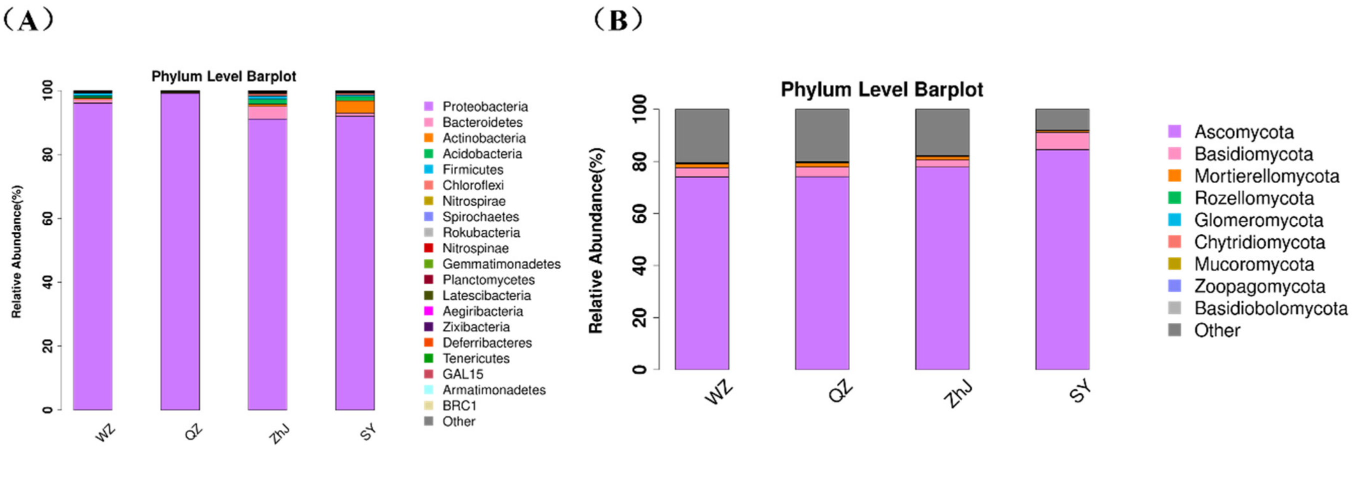

Figure S4. The relative abundance of endophytic bacterial (A) and fungal (B) communities of four populations of *W. trilobata* at the phylum level.

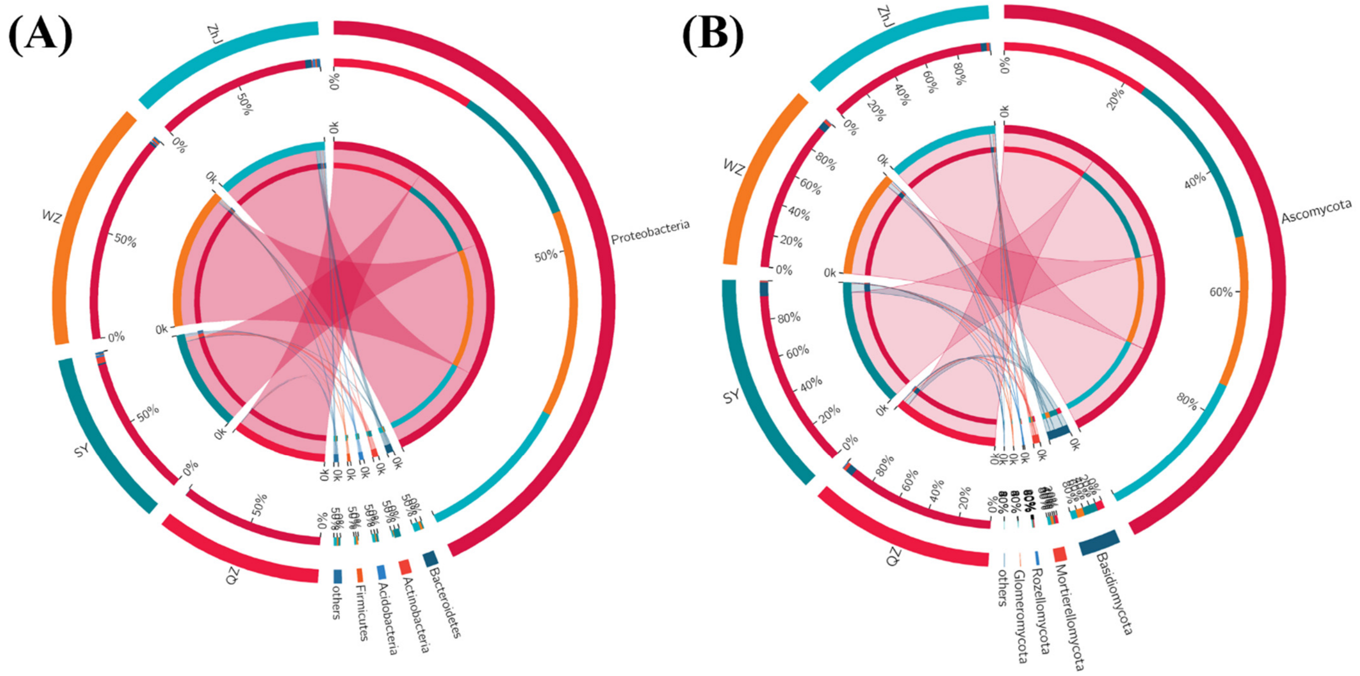

Figure S5. Circos analysis of endophytic bacteria (A) and fungi (B) in four populations of *W. trilobata* at phylum level.

Table S1. Sequencing evaluation of bacterial and fungal community in four populations of *W. trilobata*.

	Group	Clean_tags	Effective_tags	OTUs	Goods_coverage
**Bacterial**	WZ	201995	194982	478	0. 998
	QZ	249494	241884	268	0. 999
	ZhJ	280307	267232	860	0. 997
	SY	199289	191401	662	0. 998
**Fungal**	WZ	365075	348583	2820	0. 998
	QZ	301155	286795	2857	0. 998
	ZhJ	333131	316938	2947	0. 998
	SY	379471	369108	2483	0. 997
